# The use of motion tracking technology to support an exercise intervention for children and young people with cancer: perspectives of users and healthcare professionals in the FORTEe trial

**DOI:** 10.3389/fped.2026.1879166

**Published:** 2026-07-08

**Authors:** Alba Solera-Sanchez, Hayley Marriott, Stanley Windsor, Kim Straun, Marie A. Neu, Elias Dreismickenbecker, Francesca Lanfranconi, Linda Peli, Joachim Wiskemann, Nikolai Bauer, Filippo Spreafico, William Zardo, Tobias Baader, Peter Wright, Joerg Faber, Eila Watson, on behalf of the FORTEe Consortium

**Affiliations:** 1 School of Sport, Nutrition and Allied Health Professions, Faculty of Health Science, and Technology Oxford Brookes University, Oxford, United Kingdom; 2 School for Policy Studies, University of Bristol, Bristol, United Kingdom; 3 Childhood Cancer Center Mainz, University Medical Center of the Johannes Gutenberg-University Mainz, Mainz, Germany; 4 Centro Maria Letizia Verga, Fondazione Monza e Brianza per Il Bambino e La Sua Mamma, Monza, Italy; 5 Department of Medical Oncology, Working Group Exercise Oncology, Heidelberg University Hospital and National Center for Tumor Diseases, a Partnership Between German Cancer Research Center and University Medical Center Heidelberg, Heidelberg, Germany; 6 Oncology Unit, IRCCS Istituto Giannina Gaslini, Genoa, Italy; 7 Pediatric Oncology Unit, Fondazione IRCCS Istituto Nazionale dei Tumori, Milan, Italy; 8 Pixformance Sports GmbH, Berlin, Germany; 9 Oxford Institute of Applied Health Research, Faculty of Health Science, and Technology, Oxford Brookes University, Oxford, United Kingdom

**Keywords:** cancer care, childhood cancer, exercise, motion tracking technology, supportive technology

## Abstract

**Introduction:**

Technological advances provide new approaches to improving oncology care. Research indicates that supportive technological tools can benefit children with cancer, and integrating technology into exercise-based interventions may enhance physical activity and reduce fatigue. The FORTEe clinical trial incorporated a motion tracking device (Pixformance) within a broader, individualised exercise intervention for children and young adults (CAYA) with cancer. The device used an inbuilt camera, avatar-led demonstrations, and real-time feedback on exercise form. This FORTEe sub-study explored the perspectives of CAYA with cancer and exercise and healthcare professionals (EHCP) on using this motion tracking technology.

**Methods:**

CAYA aged 4–21 years and EHCP from five participating centres were eligible. Experiences and perceptions were explored through semi-structured interviews with CAYA and an anonymous online survey for EHCP. The CAYA interviews and open-ended text from the EHCP survey were analysed using an inductive content analysis approach.

**Results:**

Ninety CAYA (mean age 11.1 ± 3.7 years; 44.4% female) participated in interviews and 33 EHCP completed the survey. CAYA found the device a novel and useful addition to conventional training, appreciating its variety of exercises and real-time guidance. Some technical issues affected motion detection consistency. EHCP recognised benefits for supervised exercise but highlighted challenges with transport and offline use. CAYA also suggested adding gamification features such as rewards, avatar customisation, and competition.

**Conclusion:**

The motion tracking device was well received by both CAYA and EHCP. Findings suggest this technology could complement conventional exercise interventions and provide insights for optimising efficiency, motivation, and user experience in clinical settings.

**Clinical Trial Registration:**

ClinicalTrials.gov (NCT05289739).

## Introduction

Over recent decades, advances in diagnostic technologies and treatment modalities have significantly improved survival rates for childhood cancer patients (1). Nevertheless, children and young people still experience prolonged and intensive treatments that often lead to side-effects such as pain, fatigue, nausea and loss of physical function (2, 3) with a negative impact on health-related quality of life (HRQoL) (4). Emerging evidence suggests that exercise interventions in paediatric oncology can have beneficial effects, helping to improve HRQoL, reduce fatigue, and restore physical function (5, 6). Despite these positive effects, patients report a range of factors that hinder participation in physical activity (PA) or exercise during treatment. Commonly reported barriers for PA include fatigue, shortness of breath (7), decreased fitness and physical function (8), uncertainty about safe and appropriate exercises (9), and low motivation and mood (10). Additionally, parental concerns often compound these barriers, with fears of injury, worsening fatigue, or increased risk of infection, especially for immunocompromised children (11), further limiting engagement in PA.

The hospital environment also complicates efforts to promote PA, with hospital infrastructures often ill-suited to support mobility and autonomy during inpatient care (12). Consequently, sedentary behaviour is frequently observed among children undergoing cancer treatment (13, 14) Addressing this issue is crucial, and technological advances offer innovative ways of supporting exercise within oncology care (15). Supportive technologies include mobile apps, web-based programmes (16, 17) and active video gaming (18). Encouragingly, previous literature has shown that children with cancer respond positively to such technologies, reporting them as enjoyable, and valuable (19, 20). Moreover, studies using technology to implement exercise interventions in children have shown positive outcomes, with patients increasing PA levels, meeting age-related PA recommendations (21), and experiencing reducing side-effects such as cancer-related fatigue (22). Parents have also emphasised the need for improvements to ward environments to encourage exercise, such as freely accessible equipment, engaging activities, and technologies that promote fun and active play (23).

One such technological innovation is *Pixformance*, a device that provides real-time feedback on exercise form to enable safe executions through motion tracking technology (24).

This study explores the perspectives of children and young people (CAYA) with cancer and exercise and healthcare professionals (EHCPs) involved in the FORTEe clinical trial (25), on the use of the motion tracking device within an individualised exercise programme and considers its broader potential for integration into paediatric oncology care.

## Materials and methods

### FORTEe trial design

The motion tracking device (*Pixformance)* (see below for description) was used within the FORTEe cross-European, multicentre randomised controlled trial (25). The trial investigated the effects of a personalised exercise intervention in children and young people (aged 4–21 years old) undergoing cancer treatment. The inclusion criteria for the FORTEe trial are shown in Table 1.

**Table 1:** FORTEe trial inclusion criteria.

Inclusion criteria
Diagnosis of cancer, according to the ICCC^a^ (incl. Primary, secondary and relapsed disease)
≥4 to ≤21 years
Planned or started chemotherapy and/or radiotherapy
Receiving treatment at one of the FORTEe recruitment centres
The patient is assessed by the treating team as suitable to participate in the trial, e.g., due to medical or psychological reasons.
The patient (and the legal guardian) has/have sufficient knowledge of the respective national or English language

a ICCC International Classification of Childhood Cancer.

**Figure 1:**
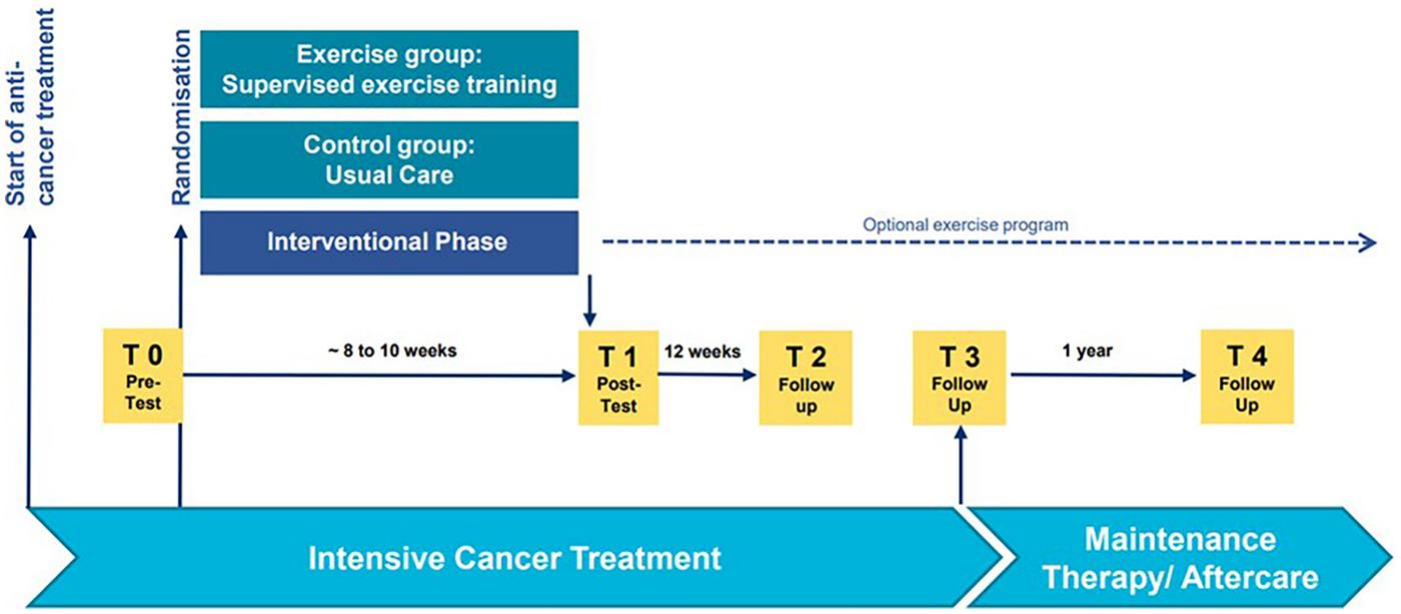
Trial timeline (25).

Participants were recruited after starting cancer treatment and underwent pre-testing prior to randomisation. Participants were randomly assigned to one of two groups: an exercise or control group. The exercise group was offered an 8–10 weeks' personalised exercise programme which included face-to-face supervised sessions, and optional virtual training, with the control group receiving routine clinical care. After the initial intervention period, both groups were offered participation in the usual exercise provision available at their childhood cancer centre (where applicable) (see Figure 1 for summary of the trial's timeline).

### Technology sub-study design

A parallel sub-project used the motion tracking technology to supplement the individualised exercise programmes, and half-structured interviews and an online survey were used to gather patient and EHCP insights. Five of the ten FORTEe recruitment centres participated in the technology-focused subproject, including one UK centre, two Italian centres, and two German centres (Appendix A). For the participants allocated to the exercise group, the motion tracking technology was offered as part of the exercise programme, and for the control group, after the intervention phase had finished (i.e., T1).

### The motion tracking device (pixoformance)

**Figure 2:**
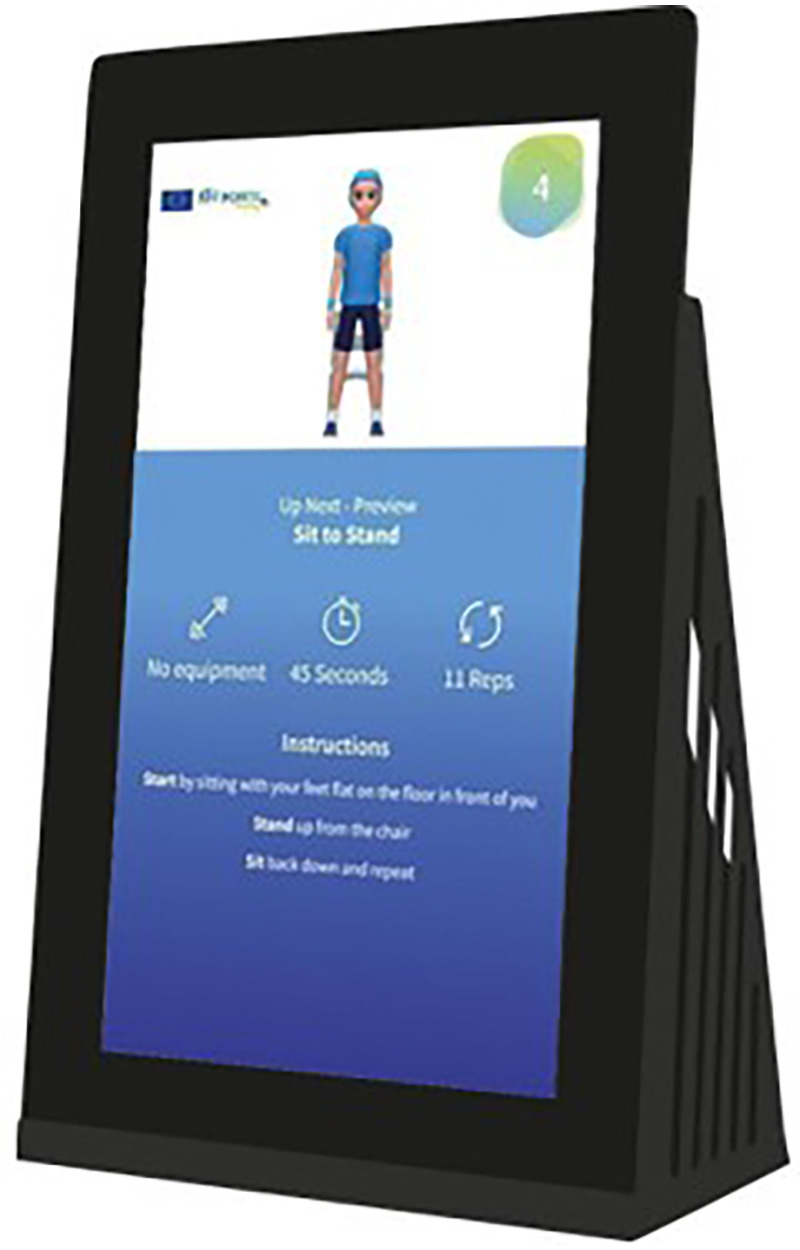
Motion tracking technology (Pixformance) with the integrated FORTEe avatar.

The motion tracking device (*Pixformance*) (Figure 2) is a free-standing station (H 129 cm/W 76 cm/D 50 cm) that provides real-time feedback on movement execution through an inbuilt camera system that tracks 25 points of the body (incl. joints). As part of the FORTEe trial, the Pixformance motion-tracking platform has been further enhanced to cater specifically to paediatric cancer patients in hospital settings. These enhancements include the integration of a child-friendly avatar that demonstrates exercises, screened exercises to ensure suitability, and a library of pre-installed workouts targeting the upper body, lower body, and core, with both standing and seated exercises available. The development of the *FORTEe* exercises and avatar demonstrations was carried out in collaboration with exercise scientists, academics, and technology experts.

The tracking system employs motion-detection technology to deliver real-time feedback through visual indicators such as arrows, circles, and live on-screen messages during exercise execution. A gamified scoring mechanism is integrated into the system, represented by a progress bar that is displayed throughout the exercise session. Scoring is derived from tracking-based analyses and reflects the extent to which the participant's movements align with the prescribed exercise patterns. The overall score varies according to the accuracy and consistency of performance. The device needs internet connection for the initial set up, and to adapt or create new exercise programmes using the online platform. However, the station can also be used offline when delivering exercise sessions. Video is not captured by the device, and no personal data is required, or stored.

The motion tracking technology was used as a supplementary training tool as part of the participants individualised exercise programme. Supervised exercise sessions were planned 3–5 times a week for 45–60 min. Exercises, duration and intensity were individualised to the patient's fitness, abilities, health condition and age. The sessions consisted of age-appropriate exercises which work on several physical fitness components such as endurance, strength, flexibility, and balance. Exercise professionals were able to create personalised workouts using an online platform or select from pre-installed FORTEe example workouts. The delivery of these sessions remained flexible, leaving specific pedagogical approaches to the clinical discretion of the exercise professionals. Consequently, instructional strategies likely differed across cohorts to accommodate varying developmental needs and goals. For younger children (ages 4–12), sessions were anticipated to favour a play-based, highly supervised format with high exercise variation to maintain engagement. Conversely, for adolescents and young adults, the approach likely shifted toward fostering autonomy, utilising the platform's real-time feedback and scoring features to promote independent self-monitoring and progression.

### Patient recruitment & data collection

Children and young adults from the five centres participating in the technology sub-project were invited to use the motion tracking technology station. While CAYA in the intervention group were encouraged to use the device, participation was optional, and no specific frequency was mandated. Control group patient participants were offered to use the motion tracking technology after the intervention period ended.

Patient participants were all invited to take part in half-structured interviews, which included asking if they had used the technology and, if so, exploring their experiences and views. The CAYA in both the intervention and control groups took part in half-structured interviews from T1 to T4. Questions specifically related to *Pixformance* were included in the interviews for the intervention group starting at T1, and for the control group starting at T2 (12 weeks after T1). Interviews were conducted by experienced researchers, face-to-face, or by phone, using a topic guide (Appendix B) that covered overall experience with the technology (hardware and software), avatar, exercise demonstrations, and available exercises and sessions.

### Exercise and health care professional recruitment & data collection

Once the intervention period was finished, FORTEe consortium EHCPs were asked to participate in an anonymous online survey that gathered their perspectives on use of the motion tracking technology. The survey explored views on the motion tracking technology set up, exercise prescription, training of other staff members, and the use of the motion tracking technology as a supportive training tool. Additionally, some questions with Likert-scale responses were included to capture perspectives on usability, usefulness as a supportive technology, patient's interaction and enjoyment with the station, and whether they would recommend it for other childhood cancer centres.

### Data analysis

The CAYA interviews and open-ended text from the EHCP survey were analysed using an inductive content analysis approach (26, 27). Initial coding of the responses was carried out independently by two researchers (AS and HM) using an inductive approach. After discussions between AS, HM and EW, a coding framework was agreed upon and subsequently applied to the dataset. Any differences in coding were discussed and resolved through consensus. Although formal data saturation was not prospectively assessed, final review of interview and survey responses did not identify any new codes or categories. The resulting codes were then organised into categories that summarised key patterns across the data. The analysis was primarily descriptive in nature and aimed to systematically capture and structure participant perspectives.

The anonymous online survey for EHCPs also included some questions which used 5-point Likert scale responses (e.g., from “strongly disagree” to “strongly agree”). Descriptive statistics, such as frequencies and percentages, were utilized to summarize the responses of EHCPs to each item. No inferential statistics were employed because of the exploratory nature of the survey.

## Results

**Figure 3:**
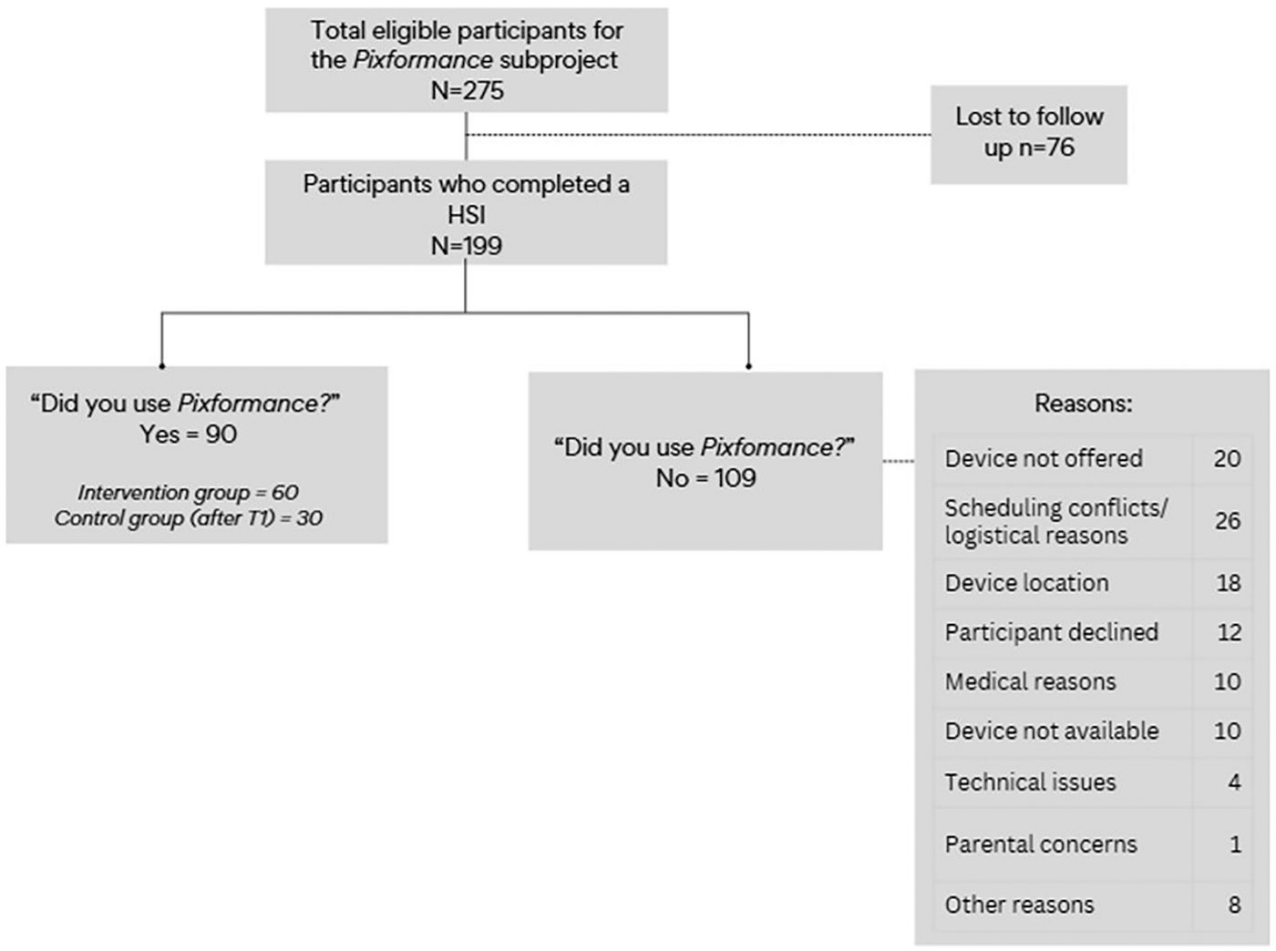
Study flowchart.

Figure 3 illustrates the inclusion of FORTEe participants eligible for the AR subproject.

Table 2 presents the demographic and clinical characteristics of CAYA who provided feedback on the use of *Pixformance.*

**Table 2:** Patients characteristics.

Participant characteristic	Value
Age, years ± SD (age range)	11.1 ± 3.7 (4–21 years)
Sex (n, %)	
Female	40 (44%)
Male	50 (56%)
Diagnosis	
Leukemia	48 (53%)
Lymphoma	17 (19%)
Malignant bone tumours	7 (8%)
Soft tissue sarcoma and other extra osseous sarcoma	6 (7%)
Neuroblastoma	5 (6%)
Renal tumours	3 (3%)
Germ cell tumours	2 (2%)
Central nervous system tumours intracranial and intraspinal	2 (2%)

With regards to EHCP, feedback from a total of 33 EHCP was collected through the anonymous online survey.

### Interview findings

#### Experience with the motion tracking technology

CAYA across all ages reported that they liked using the device and thought the technology was “very cool”, “great” and “fun”. A few CAYA reported that they felt that it was a motivator to keep active. Older participants commented that the technology and its design was both interesting and appealing, and that it was “great” being able to exercise autonomously. Many CAYA, of all ages, responded positively to the novelty of the device, and expressed it was something different to what they had previously used before. However, some mentioned that they did not like the wait between exercises (typically 10–30 s) and a few also reported they felt the novelty of the technology quickly wore off and became a “bit repetitive” and “boring”.

94.1% (16/17) of EHCPs who were involved in explaining to CAYA how to use the motion tracking technology, said that they found it easy/very easy to understand how the station worked and all of those who used it as a support in one-to-one sessions (100%; 15/15), reported finding it useful/very useful. EHCPs reported positive feedback from CAYA about the motion tracking technology and felt they “used it willingly”. 94.1% (16/17) believed CAYA found using the station enjoyable/very enjoyable. They also reported that “patients had no problems navigating through the exercises” and mentioned that it was “intuitive” and “interesting” for them. It was highlighted as a benefit that the station offers the possibility to engage children in exercise more regularly, since some CAYA are able to use it independently. EHCPs also mentioned the motion tracking technology being a helpful option “for when children are isolating for longer periods” and “(it) allows even the frailest patients to be trained”. They felt that the motion tracking technology is a good addition to conventional training. However, they felt it could not replace face-to-face training, since young patients need help to exercise with it, and others would find it boring after a while. EHCPs mentioned that CAYA opinion of the motion tracking technology tended to vary with age, and reported that “younger children did tend to lose concentration and engagement quicker than older patient participants”. The majority of EHCPs [72.7% (24/33)] reported that they would recommend this technology to other childhood cancer centres.

#### Views on the equipment

##### Hardware

The majority of CAYA reported that they appreciated the large motion tracking technology screen as it was “easy” and “clear” to see. The integrated camera was also well received and CAYA “liked that you could see yourself back” and “that it reveals my movements while doing the exercises”. A few reported that the station was not always kept in the best location, usually due to space constraints. One participant said “I did not like that everyone could see me doing exercises” and another mentioned “I did not like that you could not move it to different rooms”, highlighting issues with transportation of the device. EHCPs also noted practical challenges relating to the size of the station, where it could be placed and the difficulty moving it, the lack of Wi-Fi availability on the ward to be able to create or modify exercises through the online platform, lack of space for CAYA to exercise, and interruptions by staff or other CAYA.

##### Software

Many CAYA reflected that the real-time feedback offered via arrows and circles was a helpful addition to the device as it helped them improve their exercise form, although one younger participant reported finding the guide arrows and circles confusing.

The scoring system was generally well received by CAYA of all ages and groups. They reported finding it motivating, with some expressing that they enjoyed trying to “beat their score” and that it felt like a “challenge”. One participant, however, did comment it was unclear what they were getting points for. It was noted, however, that sometimes the tracking technology did not work well, or at all and some CAYA pointed out that when this affected their score, this became frustrating and demotivating.

EHCPs reported “the written instructions and written real-time feedback was infrequently read by children” and suggested that audio feedback might be more helpful.

EHCPs also noted that children particularly enjoyed the gaming effect implying that improving the gamification features might make the station more appealing and motivating.

#### Sessions/exercises

All CAYA agreed on the good variety of exercises offered and liked knowing they were tailored for them. However, they reported mixed perceptions about the difficulty and intensity of prescribed exercises. Some of the youngest participants felt that the exercises were too easy, or too slow, whilst others felt they were “too hard” or “just right”. Some of the older participants suggested that the motion tracking technology should have an option for them to modify the intensity, sets and repetitions of the sessions/exercises.

Some CAYA across different ages, described feeling they were exercising with the avatar as a partner, and that made the session fun and motivating. However, others reported that the avatar “was boring”, and that it “did not look like them”. There was consistent feedback from CAYA and EHCPs that the avatar should be customisable.

EHCPs also reported that the motion tracking technology offered a good variety of exercises which allowed them to create tailored and individualised sessions. Interestingly, one professional noted that including “Some more information on using weights etc. might be helpful as well”. In addition, EHCPs said that being able to add new exercises or change them without needing to be online, would be useful, since sometimes flexibility and adaptability mid-session is required.

#### Exercise demonstrations

Many CAYA expressed that they “liked” the avatar and found it “cool”, and “fun”.

The avatar demonstrations were generally considered to be helpful in showing CAYA how to do exercises correctly, however, it was also reported that this should be displayed for longer. Many CAYA expressed that they “liked” the avatar and found it “cool”, and “fun”, and they found it “cool to be able to see yourself back” when exercising as it helped them to understand and perform the exercises correctly. EHCPs agreed, adding that children and young people “liked to see themselves doing it”, they thought it was “funny” and therefore “They were happy after sessions”.

#### Suggestions for improvements

Suggestions for improvements to the motion tracking technology are summarised in Table 3 below.

**Table 3:** Suggested improvements.

Category	Example(s) quotes	Recommendation(s)
Device	“Sometimes the tracking didn't work properly and as a result the patients got a lower score, which demotivated some patients” (EHCP)	i) Improvements to technology/software
	“I think if possible, there could be improvement made to improve how transportable the devices is because currently it is very heavy and does not have wheels for example” (EHCPs)	ii) Providing solutions for transportation of the device
	“I did not like that you could not move it to different rooms” (CAYA, age 7 years)	
Additional gamification elements	“Incentive factor of collection points” “Earning coins or points would be motivating” (EHCPs)	i) Include incentives or rewards
	“I think there should be more options for other styles of avatars…. such as superheroes or cartoons” (EHCPs) “It would be nice to have customisable things like outfits or accessories” (CAYA, age 17 years)	ii) Increase Avatar customisation
	“It may also be nice to have a “ two player” option where you can take turns and play against each other” (EHCPs) “It would be good competing with the avatar” (CAYA, age 9 years)	iii) Increase competitive features
Exercise sessions	“I think you should be able to select exercises straight from the station rather than having to create them on the online platform. This can sometimes be a barrier and require increased organisation and does not allow for flexibility and adaptability mid-session.” (EHCPs)	i) Wider range of exercises and sessions without being online
	“Some advice regarding the use of weights or how exercises can be adapted would be nice.” (EHCPs)	ii) Incorporation of more information and options for adaptations of the exercises
	“The written instructions and written real-time feedback were infrequently read by children and perhaps audio feedback would be more helpful” (EHCPs)	iii) Audio feedback

EHCP, exercise and healthcare professions; CAYA: children, adolescents and young people with cancer

## Discussion

This study has demonstrated the potential of a novel, emerging technology including motion detection tracking and real-time feedback (*Pixformance*) which has been tailored to offer an exercise programme to support children and young adults with cancer during and after treatment. The motion tracking technology was well received by both children and young adults with cancer and the EHCPs and was seen as a positive adjunct to conventional training. These positive perceptions, reported by both CAYA and EHCPs, align with key constructs of the Technology Acceptance Model, which suggests that perceived usefulness and perceived ease of use are determinants of technology acceptance (28, 29). No adverse events were noted during participation. CAYA particularly appreciated tailored exercise prescriptions and also found the gamification aspects appealing and motivating.

Areas for improvement to the technology were identified, such as incorporating rewards and challenges, that are likely to enhance the utility of the motion tracking technology and similar tools.

Our findings resonate with previous literature on this topic. EHCPs saw the motion tracking technology as a useful adjunct to traditional training, although not a replacement for interaction with an EHCP. This finding is consistent with the Self-Determination Theory, which emphasises the importance of meaningful interpersonal connections for sustaining motivation and engagement (30). CAYA with cancer spend a considerable amount of time in isolation, or alone with their parents or carers, often missing out on social interactions (31). Previous studies have shown that patients, as well as survivors, value social and shared activities, especially with an exercise professional, since positive experiences during sessions (e.g., encouragement, recognition, pride) may positively influence self-image and self-esteem (32, 33). Additional literature investigating perceptions of childhood cancer survivors' caregivers showed that they also felt that human support should not be replaced by technology, since supportive technology lacks a sense of humanity, personalisation, empathy, and skills to establish meaningful and deep conversations (34).

EHCPs highlighted that a benefit of the motion tracking technology is that it allows the CAYA to exercise independently, although within a supervised environment. Previous evidence in children with chronic diseases highlights how important it is for children to take the lead in a role or even doing a task autonomously (35). For them, performing a task autonomously represents a goal to achieve, and can be much more meaningful than passively engaging in activities (35). Literature shows that for children with cancer, not being exclusively dependent on their parents and being capable to do tasks on their own, is important and meaningful (32). Insights from the current study underline this by demonstrating that patients valued being able to navigate, interact and perform the exercises independently. These findings may also be understood through the Self-Determination Theory, which suggests that sustained motivation is fostered through the satisfaction of three fundamental psychological needs: autonomy, competence, and relatedness (30).

The current size of the motion tracking technology station, coupled with the often limited space in a ward setting was considered a challenge among both EHCPs and CAYA in this study. Whilst some centres had adequate space, not all had an ideal area to use and store the station. The importance of adequate space and hospital infrastructure has been reported by Grimshaw et al. (2020) highlighting that poor ward infrastructure can negatively impact PA levels, in terms of access to equipment, age-appropriate facilities, and the availability of specialised staff (23). From the perspective of the Unified Theory of Acceptance and Use of Technology (UTAUT), these findings relate to *facilitating conditions*, which describe the organisational and environmental resources that support technology use (29, 36)While the development of technologies and equipment is important, hospital infrastructure should be considered and addressed by relevant stakeholders, to increase accessibility of such devices. Without appropriate and positive implementation of supportive technologies within clinical settings, it is unlikely the end user will experience the full scope of potential benefits.

The novelty of motion-detection and real-time feedback integrated within the motion tracking technology was valued by both CAYA and EHCPs and considered helpful to support both exercise form and motivation. It has been noted in previous literature that visual support for PA in children with chronic conditions usually results in positive experiences, increasing children' s understanding of the task to perform and being more appealing for the child (37). Aligning with the UTAUT theory, these findings reflect positive *performance expectancy* (29, 36), whereby users perceive the technology as useful in supporting exercise participation and exercise performance/execution (29, 36). However, in this study some technical limitations occasionally affected the consistency of the motion detection technology. Previous evidence in children and young people with chronic diseases showed that technical issues or complexities in digital health resources were the main barriers for its use (38). Within UTAUT, such issues may negatively influence *effort expectancy* (29, 36)*,*by reducing perceptions of ease of use and reliability. Those same technical issues might have affected the willingness of the CAYA towards consistent use of the motion tracking technology and aligned with EHCPs' feedback about the novelty of the technology wearing off relatively quickly, and thus the station being perhaps more aligned to use within sub-sessions as a training tool rather than a complete alternative to supervised, professional-led sessions. In addition, the tracking system was intermittently less reliable, which might have affected the real-time feedback.

When it comes to the implementation of exercise, evidence has reported that supervision at all times, preferably from exercise professionals, physical therapists, and sometimes from parents and sport coaches will be needed in this population (39).

Individualised exercise prescription has been shown as an effective way to support children and young people with cancer to maintain PA behaviour and limit sedentary time without posing risks (40). However, a key factor for this is a personalised approach, adapting each session and exercise to CAYA age, abilities, clinical condition and even their specific ability on the day (41). Therefore, real-time adjustments based on a patient's condition or mood, are crucial (42). This requires multi-disciplinary input. ECHPs also suggested that an additional option to further modify the load of sessions would be useful.

EHCPs reported that the FORTEe avatar was an appreciated feature especially for younger children, and it made the sessions fun and motivating. Evidence from Thorsteinsson et al. (2019) reported that exercise participation in childhood cancer patients could be positively influenced by the opportunity of having a training partner and their support during exercise (43). However, CAYA and exercise professionals agreed on the need for avatar customisation. Research with children and young people with chronic conditions that aims to understand why these users might prefer customisation in their mobile health apps is lacking (44). Nevertheless, research in adults' underlines that allowing users to customise the content and the features of the app/technology they are engaged with, offers them control and an opportunity to personalise it, which could lead to an increased sense of autonomy (44). Literature shows that there are key psychological aspects involved in this process, such as enhanced feelings of competence and relatedness, which are essential for intrinsic motivation towards a goal (45). Besides, the opportunity for autonomous customisation in supportive health care technology, leads to greater intentions for engaging in PA (43). Our results indicated that both CAYA and EHCPs also recommended adding rewards and/or incentives, which has been previously reported as a way to keep children engaged when using health and wellbeing apps (46). Previous evidence reported that this strategy helps users with motivation to keep performing daily life tasks, since they receive constant acknowledgement of their effort and accomplishments (46). Finally, CAYA and EHCPs suggested implementing challenges, such as including competitions against the avatar, or peers from the hospital. In previous evidence in young people, the addition of competitive features has also been suggested, in order to make technology more appealing (47). The desire for a competitive aspect within the app may be associated with personality traits of users. For instance, individuals with a competitive nature are constantly pursuing achievements, and therefore are more likely to be the most engaged with this enhancement. Also, the social players, who appreciate social interaction, would be interested in in-game relationships with the avatar or other peers (48). Additional literature in healthy adolescents showed that friendly competitions which lead to social interactions, could give users a sense of belonging, playing a significant role on their willingness to continue using the app (48). Nevertheless, this desire for competition may also increase stress and anxiety in some individuals (49), as well as pressure and emotional distress (48), and therefore further user-centred research is needed to better understand this mechanism. However, privacy and data safety must be considered when designing tools that allow for inter-person interaction and competition.

Future research should examine the impact of motion-tracking technology within exercise interventions on clinical, physical, and psychological outcomes. While this usability sub-study primarily explored participant experiences, further work is needed to determine whether these perceptions are associated with measurable changes in health, function, and wellbeing.

Furthermore, the broader potential of motion-tracking exercise technologies in low-resource settings warrants careful consideration. In many low and middle income countries, access to rehabilitation services and exercise professionals remains limited. Digital platforms like Pixformance could help mitigate local workforce shortages by providing participants with exercise instruction and real time feedback. However, scale-up requires careful consideration including initial equipment costs and ongoing operational constraints. For instance, participants in this study highlighted challenges regarding limited Wi-Fi availability and difficulties moving the station due to its physical size. In resource-constrained or space-limited environments, these factors underscore the importance of planning for long-term sustainability (38). Incorporating structured service-level agreements with technology providers and training IT or ward staff to handle routine maintenance may help safeguard service delivery against technical glitches and ensure continuous patient care. Crucially, the exercise content and instructions in this study were already successfully available in the native languages of the participating sites, demonstrating the platform's capacity for cross-cultural adaptation. This is vital, as most digital health interventions are designed for dominant cultural groups, limiting their appropriateness for marginalised populations (50). Providing multi-language software capabilities is essential for maximising clinical accessibility and extending the intervention's reach to meet the needs of a highly diverse patient population. While technology-supported interventions offer a highly promising opportunity for expanding paediatric rehabilitation globally, additional research is required to fully understand how such platforms can be scaled effectively across diverse, resource-variable healthcare settings.

### Strengths and limitations

This technology sub-study is part of an international clinical trial FORTEe, conducted across different countries and institutions. It is the first study performed with this kind of technology in a childhood cancer population, and with a large sample of participants. Although encouraged to respond honestly and with their own words, younger children may have found it challenging expressing their opinions during the interviews, so the data collected might have been influenced by socially accepted beliefs or family members (51), or social desirability bias (52). Furthermore, because participation was voluntary, the findings may also be subject to participation bias, as children and parents with a greater interest in the study topic may have been more likely to participate, potentially limiting the representativeness of the sample. Finally, the findings indicate that there may be slight differences in user experiences between age groups and there may also be differences related to factors such as diagnosis, sex, personality traits or levels of motivation for PA; however these subgroup differences were not explored in the present analysis, and more nuanced, in-depth research with specific user groups is needed to better understand these potential variations and to support more individualised approaches.

## Conclusion

The is the first study to evaluate the impact of a novel motion tracking device used by CAYA with cancer, using a primarily qualitative design. The study, which was conducted on a large sample of patients and EHCPs with experience of the device, indicates that the technology could be a useful support to traditional exercise sessions in CAYA with cancer. However, improvements, such as avoidance of technical issues and introduction of, gamification may be necessary to maximise engagement and user motivation. Moreover, the target population of the motion tracking technology may be broadened to children and young people with other chronic conditions.

## Data Availability

The datasets presented in this article are not readily available because the data will be available from the corresponding author upon reasonable request. Requests to access the datasets should be directed to Eila Watson, ewatson@brookes.ac.uk.

## Appendix

(A) FORTEe sub-project recruiting centres:

Oxford University Hospitals Foundation NHS Trust in collaboration with Oxford Brookes University (UK), Childhood Cancer Center of the University Medical Center of the Johannes Gutenberg-University Mainz(Germany) & Heidelberg University Hospital (Germany), Fondazione Monza e Brianza per Il Bambino e La Sua Mamma, Monza (Italy) & IRCCS National Cancer Institute Foundation (Italy)

(B) Interview questions with prompts

What did you like about the Pixformance device?

What did you not like about the Pixformance device?

Interview topic prompts

- Exercise demonstrations (human vs. avatar videos)
- Avatar design
- Pixformance scoring system
- Usability of the station
- Whether the tracking and feedback worked properly
- Exercises available
- Duration of workouts
- Intensity (RPE)
